# MAP0004 provided consistent migraine pain relief even after repeated administration

**DOI:** 10.1186/1129-2377-14-S1-P190

**Published:** 2013-02-21

**Authors:** S Kori, B Lu, E Connors, X Li, D Kellerman

**Affiliations:** 1 MAP Pharmaceuticals, USA

## 

Oral tablets are the predominant route of administration for the acute treatment of migraine. Gastric Stasis (GS) is commonly associated with migraine, and can significantly alter the rate of intestinal absorption of an oral tablet, leading to inconsistent response to the administered drug. The Tmax of a triptan administered as a tablet can vary from 25-120 min. MAP0004, an investigational drug that delivers dihydroergrotamine (DHE) systemically via the lungs using the TEMPO® inhaler, bypasses the gastrointestinal tract. Consequently, GS is likely to have no effect on the absorption of drug into the bloodstream. MAP0004 administration consistently achieves a DHE Tmax between 7-12 min. A consistent Tmax, however, does not necessarily represent a consistent clinical response. A retrospective analysis was undertaken to determine whether pain relief rates were consistent across the 1st, 5th, 15th and 25th headache treated with MAP0004.

A total of 153 subjects within the open label, long term safety study who had at least 25 qualifying migraines were analyzed. Pain relief at 2 hours was seen on an average of 54.1%, and there was no significant difference when comparing subsequent migraines to the first qualifying migraine or comparing the 1st, 5th, 15th and 25th all together. Similarly, analysis of pain free values at 2 hours (average=24%), sustained pain relief from 2-24 hours (average=38.4%), and sustained pain free values from 2-24 hours (average=16.7%) were not statistically different from the first qualifying migraine or comparing the 1st, 5th, 15th and 25th all together. In this retrospective analysis, MAP0004 provided a consistent and similar response rate in treating an episodic migraine attack, whether it was the 1st, 5th, 15th or the 25th headache treated.

## References

[B1] Aurora SK Silberstein SD Kori SH MAP0004, Orally Inhaled DHE: A Randomized, Controlled Study in the Acute Treatment of Migraine (301) Headache 2011 14 507 517 10.1111/j.1526-4610.2011.01869.x 21457235

